# The ubiquitin–proteasome system and proteostasis in the cochlea and sensorineural hearing disorders

**DOI:** 10.1186/s43556-026-00616-z

**Published:** 2026-10-13

**Authors:** Yiming Zhong, Bing Kong, Jinju Xu, Weiyi Huang, Yi Pan, Yuenan Liu, Mingliang Xiang, Bin Ye, Yilin Shen

**Affiliations:** 1 Department of Otolaryngology & Head and Neck Surgery, Ruijin Hospital, Shanghai Jiao Tong University School of Medicine, No. 197 Ruijin 2Nd Road, Shanghai, 200025 China; 2 Shanghai Key Laboratory of Translational Medicine On Ear and Nose Diseases, No. 227 South Chongqing Road, Shanghai, 200025 China; 3 Ear Institute, Shanghai Jiao Tong University School of Medicine, No. 227 South Chongqing Road, Shanghai, 200025 China; 4 Department of Audiology & Speech-Language Pathology, College of Health Science and Technology, Shanghai Jiao Tong University School of Medicine, No. 150 Ruijin 2Nd Road, Shanghai, 200025 China

**Keywords:** Ubiquitin–proteasome system, Sensorineural hearing loss, Inner-ear proteostasis, Ubiquitin ligase E3

## Abstract

The ubiquitin–proteasome system (UPS) is the major machinery for intracellular protein degradation, playing an indispensable role in maintaining proteostasis across all tissues. Sensorineural hearing loss affects billions of people worldwide and is characterized by the irreversible degeneration of cochlear hair cells and spiral ganglion neurons. However, effective treatments remain unavailable. Although UPS dysfunction has been increasingly recognized in aging and neurodegenerative diseases, its specific contributions to cochlear development, homeostasis, and hearing disorders have received relatively limited attention. This review systematically summarizes the roles of UPS-mediated proteostasis in the auditory system. Major UPS related components including E1/E2/E3 ubiquitin ligases, deubiquitinating enzymes and proteasome subunits were discussed in this review. A comprehensive summary of the importance of UPS in cochlear development and the maintenance of inner ear auditory function has been made. Various UPS molecules and their potential pathogenic mechanisms or related signaling pathways are summarized in different types of hearing loss, including congenital hearing loss, age-related hearing loss, noise-induced hearing loss, and drug-related hearing loss. This review first highlights the tissue-specific functional divergence of UPS among cochlear structures. It also proposes the UPS as a central regulatory hub linking multiple pathological pathways in hearing loss including mitophagy, autophagy, oxidative stress, DNA damage repair, endoplasmic reticulum (ER) associated degradation, and NF-κB mediated inflammation. Finally, the emerging therapeutic strategies that target UPS components to restore cochlear proteostasis and hearing loss are discussed. These insights lay a solid foundation for developing UPS-targeted interventions for sensorineural hearing loss, in order to achieve precise treatments according to cochlear cell-specific ubiquitinomics in the future.

## Introduction

According to the latest World Hearing Report, nearly 2.5 billion people will experience hearing impairment by 2050 [1]. Sensorineural hearing loss (SNHL), the most common form of hearing loss, is caused by dysfunction of the cochlea, auditory nerve, or central auditory processing pathways [2]. Major contributors to hearing degeneration include ototoxic drugs, aging, and noise exposure. Among these, age-related hearing loss is the most prevalent, followed by noise-induced hearing loss (NIHL) [3]. Hearing loss caused by ototoxic drugs is often irreversible. Aminoglycoside antibiotics have been associated with hearing loss rates of up to 33%, while furosemide therapy causes hearing loss in approximately 6–7% of patients [4]. Platinum-based chemotherapy drugs have even higher ototoxicity, with an incidence ranging from 20 to 70% [5], and the situation is more serious in children [6]. Congenital hearing loss affects approximately 1–3 per 1,000 newborns worldwide [7]. Multiple pathological mechanisms contribute to the onset and progression of SNHL, including genetic variants, immune inflammatory responses, oxidative stress, mitochondrial dysfunction, and impaired autophagy [8–11]. Most types of SNHL are permanent because cochlear hair cells and spiral ganglion neurons have very limited regenerative capacity [12]. Auditory impairment is also closely associated with depression, cognitive dysfunction, and other disorders [13]. However, therapeutic options are extremely limited. To date, no FDA-approved drug is available to reverse or broadly treat SNHL. Sodium thiosulfate is currently the only approved drug for reducing the risk of cisplatin-induced ototoxicity in pediatric patients with localized, non-metastatic solid tumors [14]. Existing treatments mainly rely on hearing amplification devices or cochlear implants, and cannot reverse the progression of SNHL [15]. A recent study has reported breakthrough progress in gene therapy for autosomal recessive deafness type 9 (DFNB9) caused by *OTOF* gene mutations [16]. This achievement marks a major step forward for gene-targeted therapy for SNHL. Thus, a deeper understanding of the molecular mechanisms underlying SNHL is essential for facilitating the development of etiology-based gene therapies.

**Fig. 1:**
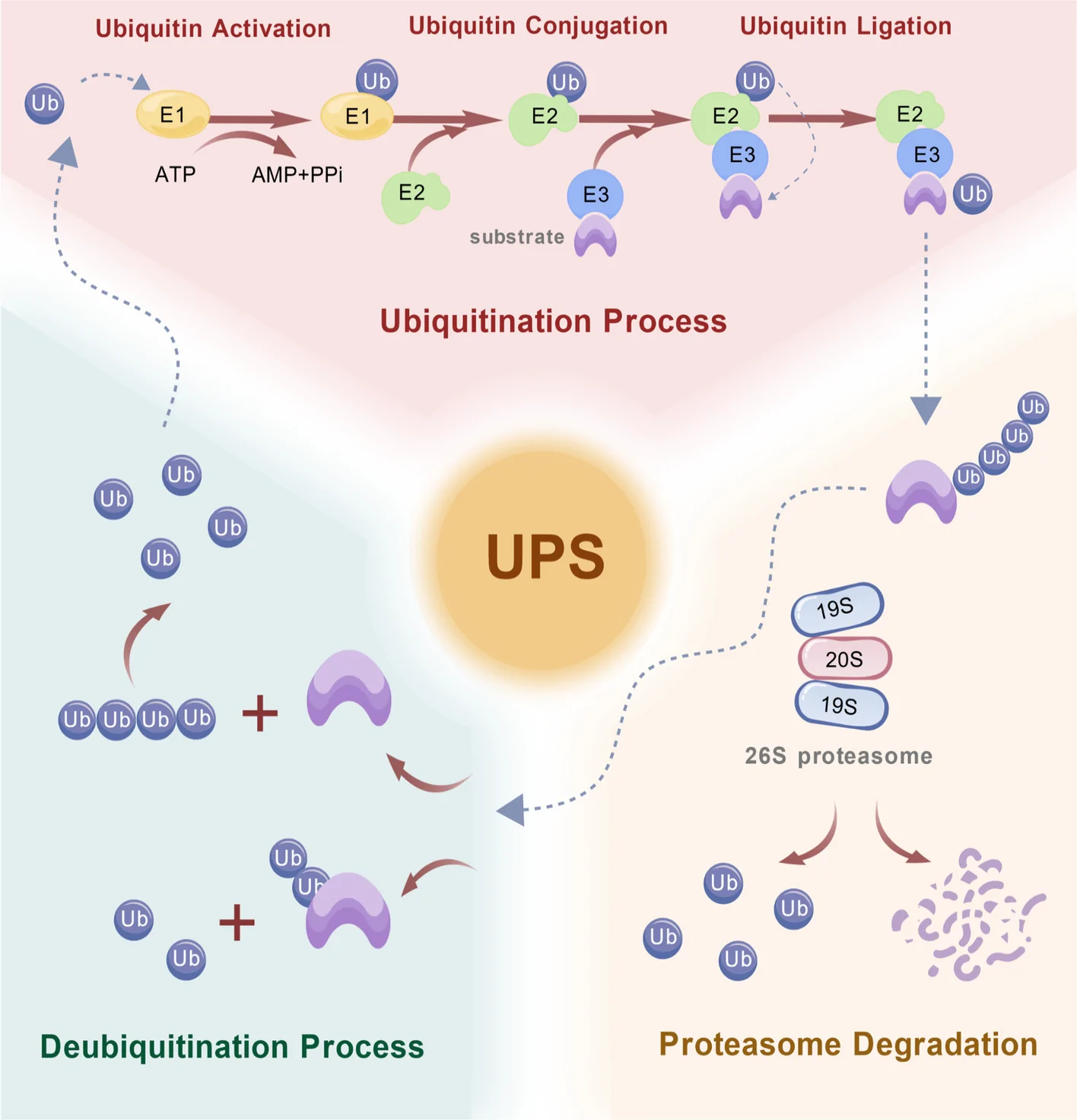
The diagram of ubiquitin–proteasome system. The process of ubiquitination is composed of ubiquitin-activating enzyme E1, ubiquitin-conjugating enzyme E2, and ubiquitin ligase E3. The deubiquitination process is also involved in the balance of protein degradation. The figure is created using BioGDP.com [31]

Protein homeostasis (proteostasis) refers to the coordinated regulation of protein synthesis, folding, transport, and degradation within the cell [17]. The clearance of abnormal proteins is often mainly mediated by the ubiquitin–proteasome system (UPS) or autophagy. Among them, the UPS accounts for up to 90% of intracellular protein degradation [18], making its components potential therapeutic targets for various diseases. The UPS comprises an enzymatic ubiquitination cascade and 26S proteasomes, which recognize and degrade ubiquitin-tagged substrate [19] (Fig. 1). Ubiquitin is a small protein of 76 amino acids, whose function is to target its substrates to the proteasome for the degradation of misfolded/unfolded protein [20]. Ubiquitination is the second most common post-translational modification [21]. It plays a crucial role in the modulation of cellular survival, proliferation and differentiation. The process of ubiquitination is primarily mediated by a multi-enzyme cascade involving the E1 ubiquitin-activating enzyme, E2 ubiquitin-conjugating enzyme, and E3 ubiquitin ligase [22]. E3 Ubiquitin ligase, the most numerous and structurally diverse member in the ubiquitination reaction system, can interact directly with substrates or through auxiliary proteins. They play key roles in determining the specificity of ubiquitin-mediated substrate selection [23]. Based on structural and biochemical properties, E3 enzymes are mainly divided into several families according to their domains [24, 25]. Ubiquitin itself contains eight potential linkage sites, including seven lysine residues (K6, K11, K27, K29, K33, K48, and K63) and the N-terminal methionine residue (M1) [26]. Among these, K48-linked polyubiquitination is the main linkage pattern regulating intracellular protein levels. It primarily marks proteins for recognition and degradation by the 26S proteasome [27]. K63-linked polyubiquitination is another major ubiquitin modification type. It is mainly responsible for nondegradative processes such as protein transport, DNA repair, and protein kinase activation [28]. Ubiquitination is a reversible process. The balance of protein degradation depends on both ubiquitination and deubiquitination. Deubiquitination is catalyzed by deubiquitinating enzymes (DUBs) to remove connection between ubiquitins or remove ubiquitins from substrates [29]. Disruption of the balance between ubiquitination and deubiquitination has been implicated in a broad range of pathological conditions, including aging, tumors, and various neurodegenerative diseases [30].

The role of the UPS in sensorineural hearing loss deserves greater attention. The sensory cells in the cochlea including hair cells and spiral ganglion neurons have limited regenerative capacity. Their long-term survival and function depend heavily on the precise maintenance of proteostasis. The UPS plays a critical role in the auditory system across different stages, from inner ear development to aging process. Therefore, this review is the first to systematically summarize the roles of protein homeostasis and the function of UPS in inner ear development and various forms of SNHL. It combines the latest studies on the UPS in central neurodegenerative diseases, which can help to better elucidate the dynamic changes and functions of UPS components in the inner ear. Furthermore, the underlying molecular mechanisms and potential common cross-pathways involved in SNHL are explored deeper. Finally, the feasibility and therapeutic potential of UPS-targeted interventions for the SNHL are discussed.

## The UPS participates in aging and neurodegenerative diseases

Given that SNHL shares several features with neurodegenerative disorders, and the degenerative pattern of the cochlea shows similarities to neuronal degeneration in the central nervous system [32], it is useful to review the role of UPS in aging and neurodegenerative diseases. This may provide an important framework for understanding how UPS dysfunction contributes to SNHL.

Many neurodegenerative diseases including Parkinson's disease (PD), Huntington's disease (HD), Alzheimer's disease (AD) and amyotrophic lateral sclerosis (ALS) are characterized by pathological accumulation of insoluble protein aggregates [33]. Impaired clearance of these abnormal aggregates is often relevant to UPS dysfunction [34]. Aging is also associated with the accumulation of ubiquitinated protein aggregates, which can inhibit the activity of the 26S proteasome activity. Thus, compromised cellular clearance of damaged proteins during aging could further exacerbate the proteotoxic effect and thereby contribute to the progression of aging. Given the age-related decline in UPS function, enhancing ubiquitin-dependent proteolysis may have beneficial effects on longevity [35]. This can be achieved by modulating the expression or activity of ubiquitination-related enzymes or the proteasome. For example, depletion of the E3 ubiquitin ligase RLE-1 in C. elegans extends lifespan by increasing the expression level of its target protein DAF-16 [36]. In Drosophila, overexpression of E3 ubiquitin ligase Parkin extends lifespan. In mammals, Parkin overexpression delays hallmarks of aging in several tissues and cell types [37]. Conversely, inactivation of the ubiquitin-activating enzyme E1 shortens the adult lifespan of fruitflies [38]. Additionally, elevated proteasomal capacity can also extend lifespan in multicellular organisms [39, 40].

In AD, the aggregation of amyloid-β (Aβ) and hyperphosphorylated tau protein inhibits proteasome activity, leading to dysfunction of the UPS [41]. Enhancement of Parkin expression and its downstream mitophagy pathway protects against neuronal degeneration [42]. Mutations in the E3 ubiquitin ligase Parkin prevent effective ubiquitination of its substrates, thereby impairing the timely clearance of abnormal proteins, and leading to early-onset or juvenile PD [43]. In peripheral neurodegenerative diseases such as ALS, large amounts of hyper- phosphorylated and ubiquitinated TDP-43 protein aggregates are found in motor neurons [44]. Lycorine has been shown to improve functional recovery in ALS model mice through promoting TDP-43 degradation by UPS. Zinc and ring finger 1 (ZNRF1), an E3 ubiquitin ligase expressed in neurons of both the central and peripheral nervous systems during development and adulthood, can be activated by oxidative stress. Activated ZNRF1 promotes the degradation of its downstream target protein Akt. Reduced expression of Akt can activate GSK3β and further cause neuronal degeneration [45]. The visual system shares several features with the auditory system, and extensive research has confirmed the important role of the UPS in retinal pigment epithelial cells and visual neurons. Glaucoma is a progressive neurodegenerative disease characterized by the loss of retinal ganglion cells and subsequent visual field impairment. Altered ubiquitin signaling is related to glaucoma pathogenesis [46]. In age-related macular degeneration, increased expression of heat shock proteins (HSPs) is closely related to the UPS activity [47]. Proteasome inhibition and the accumulation of ubiquitinated proteins can activate heat shock factor 1 (HSF1) [48]. Activated HSF1 then induces the expression of molecular chaperone, including HSPs, which help clear toxic protein aggregates by restoring proteostasis [49]. Moreover, dysfunction of the UPS can lead to increased expression of transcription factors such as hypoxia-inducible factor (HIF) and NF-κB in retinal pigment epithelial cells, further promoting inflammatory responses and exacerbating aging [50].

**Fig. 2:**
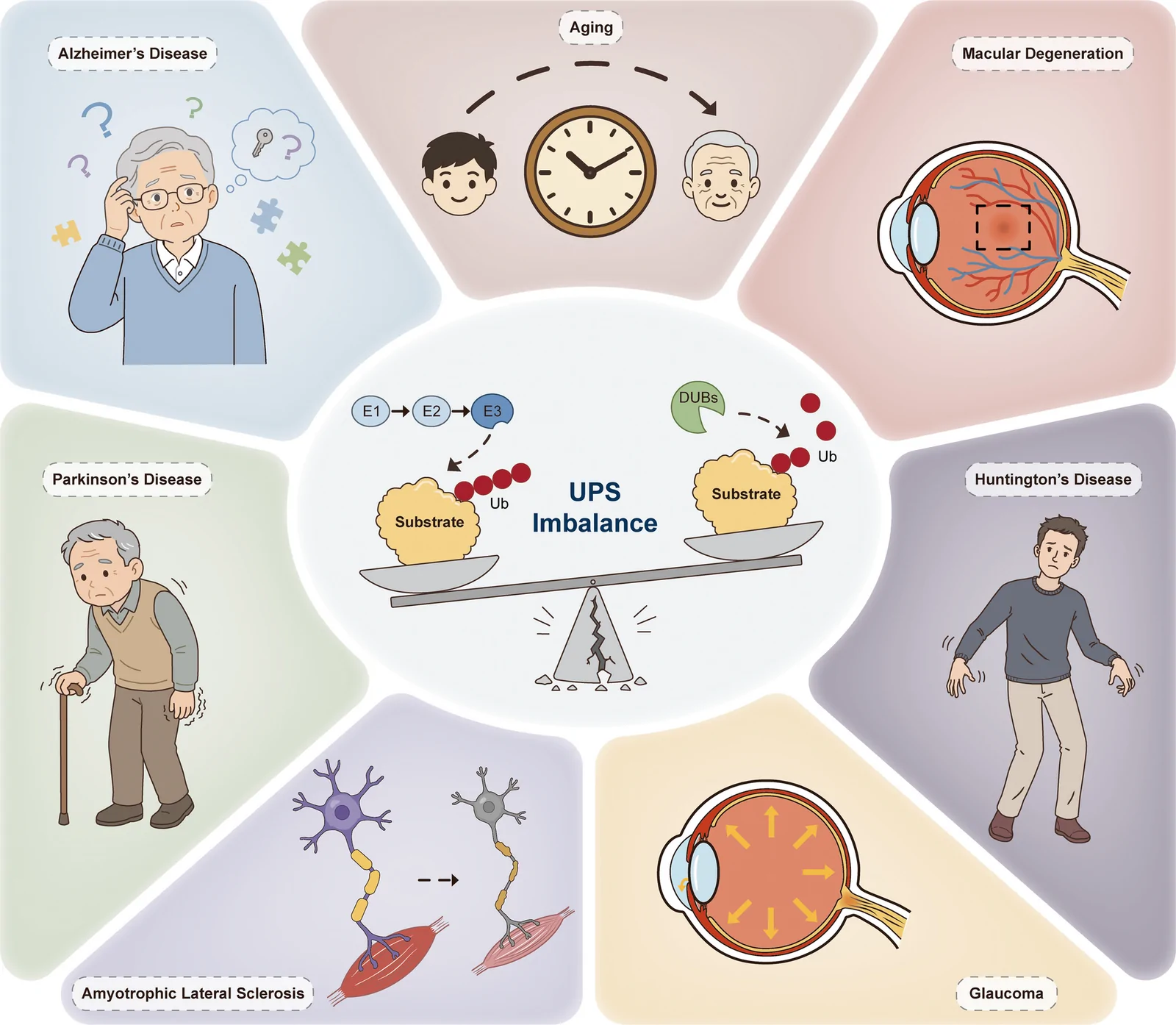
The UPS imbalance can lead to both peripheral and central neurodegenerative diseases, including aging, glaucoma, macular degeneration, Parkinson's disease, Huntington's disease, Alzheimer's disease and amyotrophic lateral sclerosis

The evidence discussed above highlights that UPS imbalance is closely involved in aging and various neurodegenerative diseases (Fig. 2). Abnormal protein accumulation, proteasome impairment, and dysfunction of E3 ubiquitin ligases are the major driving factors that lead to tissue degeneration and functional loss. Although the importance of the UPS and proteostasis in the central and peripheral nervous systems has been well recognized, their specific roles in maintaining inner ear homeostasis and their impacts on hearing loss remain poorly understood. Therefore, the following sections will focus on the role of UPS and proteostasis in the inner ear and hearing disorders.

## The importance of proteostasis in the cochlea

Proteostasis is crucial for maintaining the integrity and function of cochlear structures, including hair cells, supporting cells, spiral ganglion neurons, and the stria vascularis. Although the lifetime of most proteins in vivo is usually measured in days, a small subset of extremely long-lived proteins (ELLPs) can persist for months or even years. Studies have shown that ELLPs are highly enriched in the cochlea. Because of their extremely slow turnover, these proteins are particularly vulnerable to damage. Their dysfunction is closely associated with various forms of acquired hearing loss [51]. Sun et al. perform single-cell sequencing across 27 different cell types from the mouse inner ear and identify that loss of proteostasis and increased apoptosis are hallmarks of cochlear aging. Activation of HSP90AA1 prevents disruption of intracellular proteostasis in the stria vascularis and reduces apoptosis [52]. These findings suggest that proteostasis is important for protecting against hearing loss. However, the regulatory network of proteostasis in the cochlea is extremely complex, including protein synthesis, folding and conformational maintenance in the ER, as well as protein degradation systems (UPS and autophagy) [53]. Our team has previously published thorough reviews on the roles of ER stress [54] and autophagy-related proteostasis [55] in hearing loss. This review mainly focuses on the role of UPS-related proteostasis in the inner ear. In cultured cochlear hair cells, inhibition of protein degradation by proteasome inhibitors disrupts stereociliary bundle structure, accompanied by peroxisomal dysfunction and significant cellular damage in HEI-OC1 cells [56]. Usher syndromes are often accompanied by sensorineural hearing loss. Among them, Usher syndrome type III (USH3) is caused by mutations in the *CLRN1* gene that lead to protein instability and susceptibility to UPS degradation. In a mouse model of USH3, a small molecule compound BF844 is identified through high-throughput sequencing to stabilize misfolded CLRN1 protein. BF844 can effectively alleviate hearing loss in USH3 mice through restoring CLRN1-mediated function [57]. Thus, protein homeostasis regulated by UPS is essential for maintaining normal auditory function. In the following sections, we systematically summarize the role of UPS related molecules, including E2/E3 enzymes, DUBs, and proteasome subunits in cochlear development, differentiation, maturation, and aging process.

## The importance of UPS in cochlear development

The cochlear sensory epithelium develops through a highly coordinated sequence of events. Each step requires precise control of the abundance and turnover of key regulatory proteins within defined developmental windows. UPS is essential for cochlear development by controlling the stability and temporal activity of developmental regulators. It participates in several key events such as prosensory domain maintenance, cell-cycle exit, cell fate determination, epithelial polarity establishment, and cell survival. We further summarize the role of UPS and its underlying mechanism in various types of SNHL.

### The UPS regulation of cochlear developmental transcription factors

Atoh1 is a basic helix-loop-helix (bHLH) transcription factor required for hair cell differentiation [58]. During cochlear development, the HECT-type E3 ubiquitin ligase HUWE1 recognizes CK1-phosphorylated Atoh1 and promotes its K48-linked polyubiquitination to subsequent proteasomal degradation. Inhibition of either HUWE1 or CK1 leads to Atoh1 accumulation, resulting in the generation of extra inner hair cells and synapses. Deletion of Huwe1 during development causes early postnatal hair cell death [59]. Another important transcription factor implicated in cochlear development is the stress-responsive transcription factor p53, which is closely related to Notch signaling pathway [60]. Abnormal elevation of p53 can induce cell-cycle arrest and apoptosis, thereby compromising the survival of inner ear progenitors as well as differentiating hair cells and supporting cells [61]. The E3 ligase Mdm2 maintains low expression levels of p53 to suppress its transcriptional activity by promoting p53 ubiquitin-dependent degradation. Mdm2 is essential to maintain early inner ear progenitors’ viability. Loss of Mdm2 leads to extensive loss of hair cells and supporting cells during differentiation [62]. Another RING-type E3 ubiquitin ligase RBBP6 (also known as PACT) can facilitate Mdm2-mediated ubiquitination of p53. Knockdown of PACT in mice causes congenital hearing impairment in mice and exhibits abnormalities of the outer and middle ear, including a narrowed external ear canal, reduced middle ear cavity, and malformed ossicles. However, the cochlear structure is largely preserved, suggesting that the hearing impairment is secondary to outer and middle ear malformations [63]. In addition, RBBP6 also correlates to adult-onset deafness [64].

### The UPS regulation of cochlear developmental signaling pathways

UPS also modulates several key signaling pathways involved in cochlear development, including Notch, Wnt, and TGF-β signaling [65–67]. Notch signaling is fundamental for the establishment of the inner ear prosensory domain region [68]. The E3 ligase Mind bomb1 (Mib1) mediates ubiquitination and endocytosis of Delta ligands, a necessity for Notch activation [69]. In the Mib1 loss-of-function mutant zebrafish, the sensory cell differentiation in the ear is inhibited and the lateral wall sensory patches consist of excess and premature hair cells [70]. In the Wnt signaling pathway, the HECT-type E3 ligases Smurf1 and Smurf2 target Prickle1 for ubiquitination and degradation [71]. They are engaged in a noncanonical Wnt signaling pathway, playing key roles in inner ear signal transduction, cellular polarity, and motility [72]. Smurfs also participate in the establishment and maintenance of planar cell polarity (PCP) in hair cells. By promoting the ubiquitination and degradation of core PCP components Prickle1, Smurfs help stereociliary bundles of hair cells to maintain in a uniform direction [73]. Nevertheless, obvious PCP defects in outer hair cells are not observed in Prickle1-/- mice. Type II spiral ganglion neurons that innervate outer hair cells exhibit abnormal neurite extension [74], suggesting that Prickle1 exerts distinct roles in different structures of cochlea. Another pathway is TGF-β signaling. The RING-type E3 ligase Trim71, which is regulated by the LIN28/let-7 axis, is expressed in the early otic vesicle epithelium and is rapidly downregulated at the onset of cochlear differentiation. Trim71 restrains premature activation of TGF-β/Activin signaling by suppressing Inhba and Tgfbr2 expression, thereby maintaining prosensory cells in a proliferative and undifferentiated state. Embryonic loss of Trim71 causes early cell-cycle exit and leads prosensory cells to differentiate into hair cells or supporting cells in advance. This results in mixed congenital or sensorineural hearing loss at high frequencies [75]. Additionally, postnatal reactivation of Trim71 in the cochlea facilitates the de-differentiation and proliferation of supporting cells, as well as hair cell regenerative potential [76].

### The functions of proteasome subunits and deubiquitinating enzymes during auditory development

In addition to E3 ubiquitin ligases, subunits of the 19S regulatory particle of the 26S proteasome play critical roles in inner ear development. PSMC1, PSMC3, and PSMD12 are key subunits of the 19S regulatory particle. Among them, PSMC1 and PSMC3 are ATPase subunits, while PSMD12 is a non-ATPase subunit [77, 78]. Mutations in *PSMC1* disrupt the AAA + domain responsible for ATP binding and hydrolysis, thereby impairing proteasomal substrate recognition and degradation. These defects lead to abnormal development of inner ear neurons and semicircular canals. Patients present with sensorineural hearing loss after birth, accompanied by neurodevelopmental abnormalities and central nervous system dysfunction [77]. Forebrain *PSMC1* conditional knock-out mice display intraneuronal Lewy-like inclusions and extensive neurodegeneration, which are related to mitochondrial dysfunction [79]. Mutations in *PSMC3* cause early-onset sensorineural hearing loss with structural defects in the semicircular canals and cochlea. Affected patients show positive otoacoustic emissions during the neonatal period, but these responses rapidly disappear. Although cochlear implantation can improve hearing, language development remains delayed. In zebrafish models, loss of PSMC3 leads to obvious inner ear abnormalities including auditory hair cell death and impaired semicircular canal morphogenesis, likely through the TCF11/Nrf1 signaling pathway [78]. Mutations in *PSMD12* cause sensorineural or mixed hearing loss, often accompanied by intellectual disability, developmental delay, facial dysmorphism, and central nervous system abnormalities. PSMD12 deficiency reduces the structural stability of the 19S lid subunit and impairs proteasomal substrate recognition or degradation efficiency [80]^.^ In PD, PSMD12 has been shown to be closely associated with the NF-κB signaling pathway [81]. In autoimmune diseases models, activation of NF-κB signaling has also been observed in the context of *PSMD12* haploinsufficiency [82].

Deubiquitination is another important component of UPS during inner ear development. CYLD is a microtubule‐binding protein with deubiquitinase activity that specifically removes K63‐linked polyubiquitin chains. It is localized in neurons and cochlear hair cells. CYLD contributes to cochlear development by regulating neurite extension and the formation of hair cell-neuron connections. Loss of CYLD significantly reduces the length and proportion of neurites in developing auditory neurons and causes mild hearing loss in mice [83]. In the central nervous system, CYLD is found to promote synaptic maturation by ubiquitinating and degrading TNF receptor-associated factor 6 (TRAF6) [83]. However, CYLD may also act as a negative regulator of dopamine neuron survival in PD, and inhibition of CYLD may promote mitochondrial biogenesis, which is beneficial for the treatment of disease [84]. These findings suggest that CYLD may exert dual roles in different neuronal tissues.

Taken together, the UPS participates in inner ear development by controlling the stability, timing, and duration of key developmental regulators. It contributes largely to prosensory domain maintenance, cell fate decisions, sensory epithelial polarity and cell survival.

## Involvement of the UPS in different hearing disorders

### Congenital hearing loss

Congenital hearing loss refers to hearing impairment that is present at birth or develops in the early postnatal period. It can be unilateral or bilateral, ranging from mild to profound. It often affects language, speech, and cognitive development. Genetic factors, particularly pathogenic variants in hearing-related genes, are among the leading causes of congenital hearing loss [85]. Hearing loss is a common feature of numerous hereditary syndromes. Disruption of the UPS can lead to abnormalities of protein homeostasis and cause various hereditary syndromes including SNHL. Multiple RING-type E3 ubiquitin ligases are involved in the pathogenesis of congenital hearing loss. For example, ubiquitin protein ligase E3 component N-recognin (UBR) proteins are involved in various biological functions. Mutations in different isoforms of UBR can lead to several syndromic deafness conditions. *Ubr1* mutations lead to Notch signaling defects, thus causing Johanson-Blizzard syndrome (JBS). It is a rare autosomal recessive disorder characterized by pancreatic exocrine insufficiency, distinctive facial features, dental abnormalities, hypothyroidism, cognitive impairment and 78% of diagnosed patients exhibit sensorineural hearing loss [86]. Ubr3 plays a modulatory role in multiple sensory pathways, including the tactile, visual, auditory, and gustatory systems [87]. *Ubr3* mutations can cause a deafness-blindness disorder—Usher syndrome. In Drosophila, *Ubr3* deficiency leads to abnormal apical attachment of the scolopidia (sensory units of the auditory organ) and impairs mechanosensory transduction [88]. Further evidence suggests that Ubr3 regulates the monoubiquitination of Myosin II, thereby affecting its interaction with Myosin VIIa. Since Myosin VIIa is essential for the maintenance of hair cell stereocilia bundles, this dysregulation results in auditory deficits similar to those caused by *MYO7A* mutations [89]. *Ubr5* mutations can cause a neurodevelopmental syndrome mainly characterized by developmental delay, autism spectrum features and intellectual disability. Some patients also present hearing impairment [90]. Ubr5 can cooperate with Ubr7 to regulate Notch signaling [91]. Studies in C. elegans show that Ubr5 not only facilitates timely exit of stem fate through limiting PABP-2 protein [92], but also regulates the proteasomal degradation of SWI/SNF complex during developmental processes [93].

Rma1 is a RING-type E3 ubiquitin ligase localized to the endoplasmic reticulum membrane, responsible for the ER-associated degradation (ERAD) mediated degradation of Pendrin. Pendrin, encoded by *SLC26A4*, is an anion exchanger for maintaining pH and salt homeostasis in the kidney, lung, and cochlea [94]. *SLC26A4* mutations can cause Pendred syndrome, which is characterized by congenital sensorineural hearing loss, thyroid goiter, and abnormal organification of iodide [94]. Although the role of Rma1 in the inner ear has not yet been directly examined, we speculate that Rma1 deficiency leads to the abnormal expression of Pendrin and thereby impair its normal function. Another RING-type E3 ubiquitin ligase, RNF220, is highly expressed in the developing nervous system. Its mutations cause sensorineural hearing loss in childhood [95]. RNF220 silencing in drosophila melanogaster disrupts proper localization of lamin Dm0 (lamin B1 orthologue), promotes its aggregation, and causes a neurodegenerative phenotype [95]. Lamin B1 has been verified to be associated with cochlear development in rats [96]. In a mouse model of PD, impaired RNF220-mediated ubiquitination and proteasomal degradation of signal transducer and activator of transcription 1 (STAT1) delayed disease progression [97]. These findings indicate that E3 ubiquitin ligases exert different regulation of substrate degradation in various types of tissues.

UBE3B is a HECT domain-containing E3 ubiquitin ligase. Its mutations can cause Kaufman oculocerebrofacial syndrome (KOS), characterized by intellectual disability, hypotonia, absence of speech, and mild hearing loss [98]. Knockdown of UBE3B in human cells leads to alterations in mitochondrial morphology, decreased mitochondrial volume, and severe suppression of cell proliferation [99]. UBE3B knockout neurons exhibit increased density and aberrant morphology of dendritic spines, altered synaptic physiology, and changes in hippocampal circuit activity. The catalytic γ-subunit of calcineurin Ppp3cc shows decreased ubiquitination level and is overexpressed in neuron tissue [100]. Animal studies have revealed that *UBE3B*^*−/−*^ mice exhibit growth restriction and absence of vocalizations. The pathological changes include reduced dendritic complexity of cortical neurons and delayed synaptic development. Mechanistically, UBE3B may regulate metabolic pathways during neuronal development through promoting ubiquitin-mediated degradation of BCKDK [101].

Stub1 (CHIP) is a U-box-type E3 ubiquitin ligase. It can recognize and target the activated form of Diaph3 for polyubiquitination at lysine residues K1142/1143/1144. *Diaph3* encodes formin protein that regulates the cytoskeleton of hair cell stereocilia. Its activation will enhance polymerization of F-actin, thus leading to stereocilia abnormalities. Mutations in the 5’UTR region of *Diaph3* will result in a significant increase (≈1.5-fold) in Diaph3 protein and cause autosomal dominant nonsyndromic auditory neuropathy (AUNA1) [102]. By selectively degrading hyperactivated DIAPH3, Stub1 can prevent excessive F-actin polymerization and help to maintain normal structure and function of hair cell stereocilia. Experimental evidence shows that knockdown of *Stub1* in the mouse cochlea leads to severe disorganization of both outer and inner hair cell stereocilia, as well as degeneration of spiral ganglion neurons. Auditory tests show elevated ABR and DPOAE thresholds [103]. Additional evidence also links Diaph3 dysregulation to Notch-related cochlear defects. Selective deletion of Notch ligand Jag1 in neonatal supporting cells of mice disrupts Notch signaling and causes a hearing phenotype that resembles auditory neuropathy at 6 weeks. The mice also show defects in the inner hair cell stereocilia and increased expression of Diaph3 [104]. These findings imply that Stub1 may participate in hair cell structural differentiation via the Notch signaling pathway.

In addition to the E3 ubiquitin ligases described above, several UPS-associated regulatory proteins may also contribute to congenital hearing loss. For example, BRWD3 is a known substrate-specificity factor of the DDB1-CUL4 E3 ligase complex and is involved in the regulation of protein ubiquitination. Mutations or deficiencies in BRWD3 are associated with hearing loss, cognitive impairments, epilepsy, autism, and other neurological disorders during childhood [105]. The precise pathological mechanism of BRWD3 in auditory abnormalities remains unclear. However, available evidence suggests that BRWD3 may affect cochlear sensory epithelium development, hair cell differentiation and auditory nerve development through chromatin modification [106] or JAK/STAT signaling pathways [107, 108].

Deubiquitinations imbalances can also result in congenital hearing loss. For example, USP48 encodes a ubiquitin hydrolase, and its mutations are associated with autosomal dominant non-syndromic hereditary hearing loss. Studies have shown that missense and splice-site mutations in *USP48* can cause bilateral or unilateral SNHL. USP48 is expressed in multiple cochlear structures in human fetus, indicating its importance in early auditory development. In vitro experiments show that mutations in USP48 will disturb its enzymatic activity to hydrolyze polyubiquitin chains and consequently disrupt protein homeostasis [109]. USP48 not only directly affects neuronal development but also regulates p53 signaling via the E3 ubiquitin ligase Mdm2 [110]. In *USP48*-knockdown zebrafish, delayed development of primary motor neurons and statoacoustic neurons has been observed. Also, the number of statoacoustic neurons and acoustic startle response (ASR) are found reduced [109]. However, the structure of hair cells remains normal, suggesting that the hearing deficit may primarily arise from neurodevelopmental abnormalities. In retinal degeneration, reduced USP48 expression has been shown to activate NF-κB signaling and enhance inflammatory response [111]. *USP9X* mutations can cause Axenfeld-Rieger syndrome, which is characterized by ocular anomalies, cognitive impairment, dental defects, skeletal anomalies, hearing loss and other features [112]. Studies have shown that USP9X-mediated deubiquitination of DVL2 is necessary for the activation of the canonical WNT signaling pathway [113]. It also works with the Notch signaling pathway to regulate neural progenitor cell development synergistically [114], which suggests the possible mechanism underlying the cochlea development.

In summary, UPS and its related genes are crucial for the proper formation of auditory system during embryonic development. Dysfunction or mutations in UPS components may disrupt protein homeostasis, cell cycle regulation, neuronal development, and hair cell differentiation, thereby causing various types of congenital hearing loss, including sensorineural and mixed hearing impairments. These findings highlight the central role of UPS in cochlear development and auditory function.

### Age-related hearing loss

Age-related hearing loss (ARHL) is a bilateral and progressive hearing loss which is most pronounced at higher frequencies initially [115]. Impaired clearance of misfolded and abnormally aggregated proteins have been closely associated with ARHL [116]. UPS pathway is significantly affected by aging. Wang et al. observe a marked increase level in ubiquitinated proteins in the cochleae of 12–14-month-old C57 mice compared with young mice [117]. Similarly, substantial deposits of ubiquitin-positive granules are detected in the cochlear nuclei of aged dogs with severe hearing loss [118]. However, UPS decline does not occur in isolation. Instead, it may act together with oxidative stress, DNA damage, ER stress, impaired autophagy, and defective mitophagy to promote cochlear degeneration [54, 117, 119, 120]. Moreover, abnormalities in any component of the UPS—including E1, E2, E3 enzymes and deubiquitinating enzymes can lead to presbycusis. We will discuss them in detail as follows.

Oxidative stress is one of the major contributors to the cochlear hair cell degeneration in ARHL, primarily resulting from excessive reactive oxygen species (ROS) and diminished antioxidant defenses [121]. Keap1 is a component of the Cul3-E3 ubiquitin ligase complex that negatively regulates Nrf2. Keap1 ubiquitinates Nrf2 and targets it for degradation by the 26S proteasome. In the aged cochlea, Keap1-mediated ubiquitination and degradation of Nrf2 restrict the expression of antioxidant genes, thus leading to increased accumulation of ROS in hair cells and spiral ganglion neurons. Keap1 knockdown mice show lower ABR thresholds. Nrf2 signaling pathway is thus activated in cochlea with upregulation of antioxidant genes downstream (Nqo1, Gclc, Gclm, Txnrd1). Oxidative stress accumulation is attenuated with alleviated damage to outer hair cells and spiral ganglion neurons [122]. These findings indicate that the UPS directly influences cochlear redox homeostasis during aging process.

The DNA damage response (DDR) delays the degenerative changes in hair cells and neurons through repairing cochlear cell DNA and maintaining genomic stability. Impaired DDR is therefore considered a key molecular mechanism underlying ARHL [123]. UBA1, an E1 ubiquitin-activating enzyme that initiates the ubiquitination cascade, has been implicated in progressive SNHL. Patients with *UBA1* mutations exhibit elevated high-frequency hearing thresholds accompanied by other systemic symptoms (e.g., inflammation and hematologic abnormalities) [124]. Although the role of UBA1 in the cochlea remains unclear, studies in neurodegenerative diseases such as spinal muscular atrophy (SMA) and HD have revealed that UBA1 dysfunction disrupts the ubiquitination balance of key cell cycle proteins (including H2A and p53) and impedes DNA repair [125]. The E2 ubiquitin-conjugating enzyme RAD6B also plays a critical role in DDR through histone ubiquitination. It promotes ubiquitination of histones such as H2B and facilitate the recruitment of downstream repair factors such as 53BP1 and BRCA1 after DNA damage [126]. RAD6B knockout mice exhibit elevated hearing thresholds with age. Pathological changes in cochlea include reduced hair cells, thinning and disruption of the marginal cell layer of the stria vascularis, decreased density and vacuole-like degeneration of spiral ganglion neurons. Meanwhile, the elevated expression of DNA double-strand break marker γ-H2AX further indicates impaired DNA damage repair function [127]. RAD6B can act with RING E3 ligase RNF8 in DNA damage repair. *RNF8* knockout mice exhibit neuronal degeneration, reactive astrocytosis and cognitive decline [128]. Evidence from neurodegenerative diseases has shown that in the absence of RAD6B, the recruitment of DDR factors such as MDC1 and RNF8 remains unaffected. However, H2B ubiquitination is reduced and the recruitment of 53BP1 and BRCA1 is decreased, leading to incomplete DNA damage repair and accumulation of γ-H2AX. This can further activate p53/p21 and p16-related pathways [129], both of which are closely related to the pathogenesis of ARHL [130, 131].

Impaired mitophagy is another important pathological mechanism in ARHL. Mitophagy is a protective process that maintains mitochondrial quality control and cochlear homeostasis. Pink1 and Parkin are central regulators of mitophagy. Pink1 is a mitochondria-associated serine/threonine kinase. When mitochondrial membrane potential declines, Pink1 accumulates on the outer membrane of damaged mitochondria and serves as a damage signal to recruit Parkin [132]. Parkin is a RING-type E3 ubiquitin ligase. After phosphorylation and activation by Pink1, Parkin ubiquitinates several outer mitochondrial membrane proteins, including VDAC, MFN2, and TOMM20. These ubiquitin signals promote the recognition and capture of damaged mitochondria by autophagosomes, which subsequently fuse with lysosomes to complete mitochondrial clearance [133]. In ARHL, the expression of Pink1 and Parkin decreases in the mouse cochlea with aging. Mitochondrial ubiquitination is reduced and mitophagy is impaired, thus causing degeneration of hair cells and spiral ganglion neurons [134]. In the cellular model related to mitochondrial gene mutations associated with hereditary deafness, mitophagy pathways are upregulated. MFN2 has been verified to interact with Parkin and its ubiquitination level is increased [135].

The UPS also cooperates with ERAD to maintain protein homeostasis in the cochlea. Impaired UPS and ERAD function can lead to the accumulation of misfolded proteins and ER stress, thereby contributing to the development of ARHL [54, 117]. Mutations in *WFS1* cause Wolfram syndrome, characterized by late-onset hearing loss in patients. WFS1 is an ER membrane protein that can stabilize core ERAD-associated protein HRD1, which is an E3 ubiquitin ligase. HRD1 promotes the ubiquitination and degradation of ATF6α to inhibit ER stress [136]. In contrast, loss of WFS1 causes ATF6α accumulation and enhances CHOP-mediated apoptosis, ultimately leading to SNHL [137]. Fbx2 is another E3 ubiquitin ligase involved in ER-associated protein quality control. It is a neuron-specific F-box protein that recognizes N-linked high-mannose oligosaccharides on substrate proteins [138]. Because mannose modifications occur in the lumen of the ER, Fbx2 must recognize substrates after their retrotranslocation from the ER as part of the ERAD pathway [139]. Fbx2 participates in neuronal development and degeneration. In the cochlea, Fbx2 is particularly enriched in supporting cells and hair cells. Loss of Fbx2 causes progressive hearing loss as early as 2 months of age in the mice. Pathologically, Fbx2 deficiency induces degeneration of supporting cells in the organ of Corti at first and then affects hair cells or spiral ganglion neurons. Mechanistically, Fbx2 may protect cochlea by regulating the membrane glycoproteins. In Fbx2 knockout mice, abnormal membrane structures and increased Connexin26 (Cx26) levels have been observed [140]. These findings suggest that Fbx2 dysfunction accelerates cochlear degeneration by disrupting membrane protein homeostasis and intercellular junctions in supporting cells.

Beyond the classical mechanisms implicated in ARHL, the UPS may also contribute to cochlear aging through the newly emerging pathway in recent years such as ferroptosis. Ferroptosis is an iron-dependent form of cellular death. It is characterized by disturbed iron metabolism, lipid peroxidation, and membrane damage. It has been shown to play an important role in several models of SNHL [141]. In recent years, increasing attention has been paid to the crosstalk between ubiquitination and ferroptosis-related signaling. Nedd4 is a HECT-type E3 ubiquitin ligase that is widely expressed in the rat cochlea, including the stria vascularis, spiral ligament, organ of Corti, spiral limbus, Reissner’s membrane, and spiral ganglion neurons. Lewis et al. identify *Nedd4* as a novel candidate gene for adult-onset hearing loss [142]. Nedd4 is found colocalized with Cx26 under ER stress in mice with ARHL [143]. The expression of Nedd4 is increased in D-galactose-induced HEI-OC1 cells and in the cochlea of 12-month-old mice. D-galactose treatment also elevates intracellular Fe2+ levels, whereas the ferroptosis inhibitor ferrostatin-1 can attenuate cell injury [144]. These findings suggest that Nedd4 may participate in ARHL progression through ferroptosis-associated mechanisms.

Therefore, the UPS is closely associated with many aging-related pathways in ARHL and a wide range of molecules are involved in this process. How to regulate UPS to exert the maximal anti-aging effects in the inner ear requires further research.

### Noise-induced hearing loss

Noise-induced hearing loss (NIHL) is a form of SNHL caused by excessive sound stimulation, leading to damage in hair cells, synapses, and auditory nerves [145]. Studies have shown that the UPS is also involved in noise-induced hair cell apoptosis and necrosis. Jongkamonwiwat et al. demonstrate that high-intensity noise exposure leads to protein misfolding, cytoskeletal disruption, and synaptic damage in the cochlea. Proteomics and RNA sequencing revealed that proteasome subunits, ubiquitin-conjugating enzymes, and ubiquitinated proteins are significantly upregulated with the increasing intensity of noise. These results suggest there is a certain relationship between UPS and noise-induced proteostatic imbalance [146].

Several E3 ubiquitin ligases have been implicated in NIHL. UBE3B, which has been discussed above in the context of auditory system development, may also contribute to NIHL. In a chicken model of NIHL, UBE3B protein expression was markedly increased in the damaged basilar papilla [147]. It is crucial in maintaining mitochondrial integrity and is selected as one of the genes highly related to presbycusis [148]. Although there is a lack of studies on UBE3B in the inner ear, UBE3B is found interacting with several key synaptic proteins in the cortical regions—including ATP1A1, DOCK7, NLGN2, and STX12 in autism spectrum disorder [149]. These findings suggest that UBE3B may play an important role in protecting synaptic structures of cochlear hair cells. Parkin, another E3 ubiquitin ligase discussed above also participates in NIHL. Noise injury activates the Pink1/Parkin signaling pathway, which reduces ROS and inflammatory response to protect hair cells and synaptic ribbon structures. Impaired Parkin function leads to accumulation of mitochondrial damage, elevated ROS, and degenerative changes in hair cells, ultimately exacerbating NIHL [150].

By contrast, E3 ubiquitin ligases may also promote stress response following noise exposure. For example, the E3 ubiquitin ligase GRAIL (RNF128) may exacerbate cochlear inflammation, oxidative stress, and synaptic damage after noise exposure or aging [151]. It can regulate the stability, recycling, and lysosomal degradation of membrane receptor or inflammation-related proteins through ubiquitination pathway. GRAIL knockout mice exhibit lower ABR thresholds, reduced loss of outer hair cells and CTBP2-positive synaptic ribbons. Mechanistically, GRAIL deficiency downregulates noise-induced inflammation-related genes (CCL19, CCL21, IL25, SAA1) and the oxidative stress-related gene NOX1. The antioxidant gene GPX6 is also increased [151]. In PD, RNF128 is verified to interact with SIRT1 and promotes its ubiquitination-mediated degradation. The knockdown of RNF128 attenuates brain tissue damage in PD mice. At the cellular level, reduced mitochondrial ROS levels and increased mitochondrial membrane potential are found [152].

Beyond its involvement in noise-induced stress responses, the UPS also influences individual susceptibility to NIHL. UBAC2 is a protein containing a ubiquitin-associated domain and participates in ubiquitination process [153]. Wan et al. find that single-nucleotide polymorphisms (SNPs) in the *UBAC2* gene are significantly associated with susceptibility to NIHL. Individuals carrying the haplotype AGGA (rs3825427-rs9517701-rs7999348-rs2296860) have a higher risk of NIHL. Functional experiments show that the haplotype of rs3825427 in *UBAC2* reduces the protein expression levels, and low UBAC2 expression increases stress-induced apoptosis of hair cells [154].

USP53, a member of the largest deubiquitinase family, is highly expressed in cochlear hair cells (especially outer hair cells) and in some supporting cells. It interacts with tight junction scaffolding proteins TJP1 and TJP2 in hair cells. Mutations in *USP53* can lead to outer hair cell degeneration and increase the sensitivity to noise-induced damage in mice [155]. However, in central nervous system, USP53 is found overexpressed in patients with AD. Knockdown of USP53 prevents Aβ-induced deubiquitination of NOTCH2 and represses Aβ-induced activation of the NF-κB signaling pathway in microglia [156].

Overall, UPS mainly maintains the stability of hair cells and synapses under noise-induced stress. E3 ubiquitin ligases and deubiquitinating enzymes regulate mitochondrial quality control, oxidative stress, and inflammatory responses after noise exposure. Their dysfunction or mutations can increase the vulnerability of hair cell and synapses to acoustic injury, thereby contributing to the pathogenesis of NIHL.

### Drug-related hearing loss

Common ototoxic drugs include aminoglycosides and platinum-based antineoplastic agents. Studies have shown that accumulation of ototoxic drugs in the inner ear can lead to dysfunction and imbalance of the UPS, resulting in translational errors and misfolding of proteins [157]. The substrate-specificity factor of E3 ligase BRWD3 discussed above is a chromatin-binding protein that promotes K48-linked polyubiquitination and degradation of H3K4-specific lysine demethylase 5 (KDM5) [106]. KDM5 is an enzyme that removes tri- and di- methyl marks from H3K4, which is important for maintaining normal auditory function. Researches show that the inhibited demethylation of H3K4me2 can protect hair cells and spiral ganglion neurons against various drug related hearing loss caused by neomycin [158], gentamycin [159] and cisplatin [160]. Consistently, KDM5 inhibition attenuates cisplatin-induced hearing loss via the regulation of MAPK/AKT pathway [161].

Also, UPS-related autophagy and mitophagy are involved in drug related hearing loss (DRHL). For instance, Kim et al. found that gentamicin treatment significantly reduces the expression of the deubiquitinase UCHL1, leading to autophagosome accumulation and impaired autophagic flux. This process is accompanied by marked apoptosis of hair cells and spiral ganglion neurons. In cultured spiral ganglion neurons and HEI-OC1 cells, downregulation of UCHL1 exacerbates gentamicin-induced ototoxic damage, and is closely correlated with autophagy [162]. In the kanamycin induced deafness model, the drug rapidly enters hair cells and promotes Pink1 accumulation on the outer membrane of damaged mitochondria via RIPOR2 mediated changes in mitochondrial localization. Then Parkin-dependent mitophagy is activated. Abolishing the expression of Pink1 or Parkin protects hair cells from drug ototoxicity [163]. In cisplatin induced deafness model, reduced ubiquitinated protein level is observed in hair cells and is associated with hair cell apoptosis [164]. In cisplatin treated HEI-OC1 cells, the expression of Pink1 and Parkin is both increased. However, the knockdown of Parkin can exacerbate mitochondrial dysfunction and accelerate loss of cell viability. These findings suggest that mitophagy mediated by UPS may exert opposite effects on different ototoxic drugs.

Thus, the roles of UPS in different types of drug-induced hearing loss may have significant specificity and requires further investigation.

## The links between UPS-related mechanisms in different types of SNHL

**Fig. 3:**
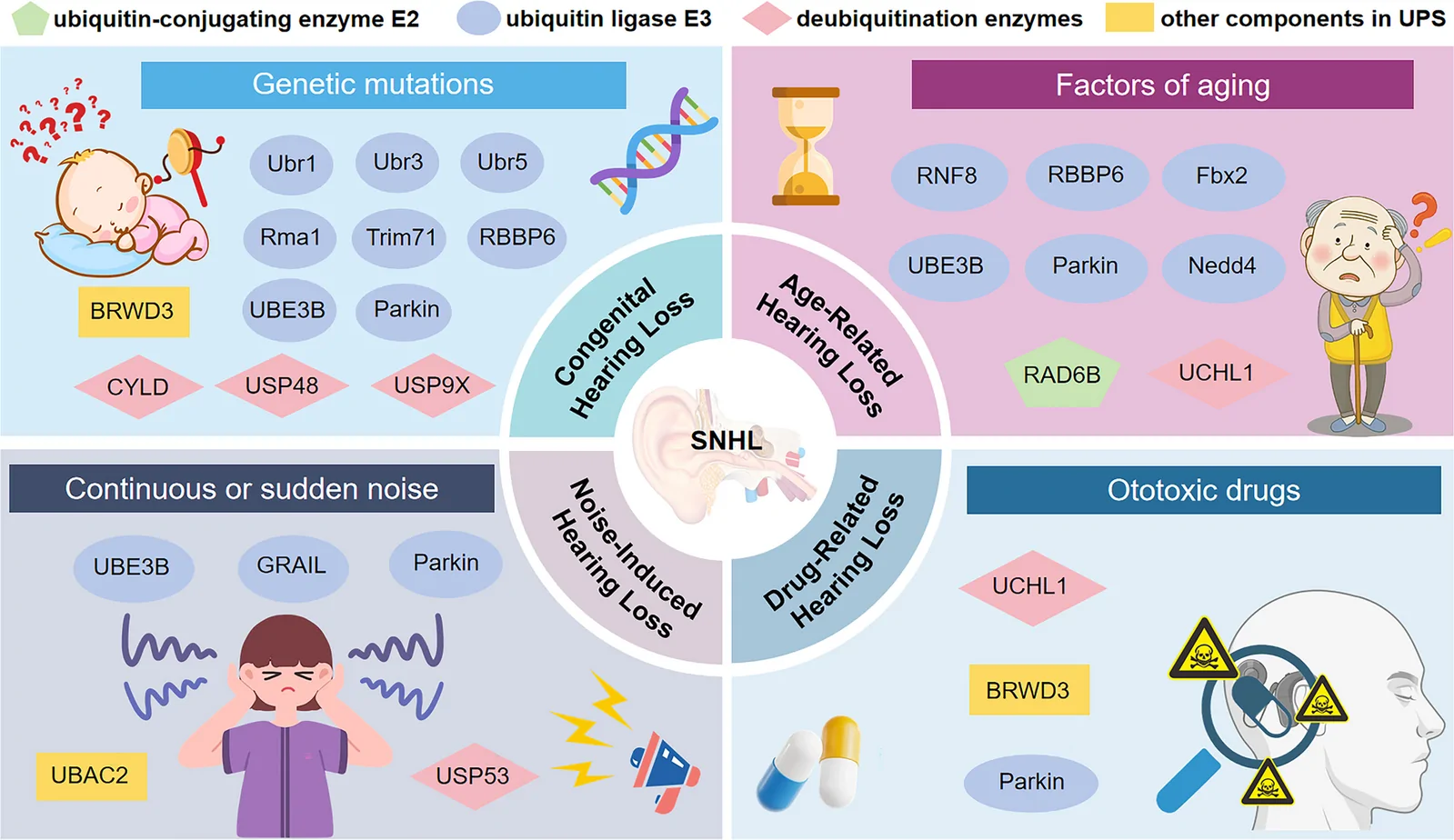
Different UPS related proteins including E2/E3 enzymes, DUBs, and other components participate in congenital hearing loss, age-related hearing loss, noise-induced hearing loss and drug-related hearing loss. Most of them are involved in congenital hearing loss followed by age-related hearing loss

**Fig. 4:**
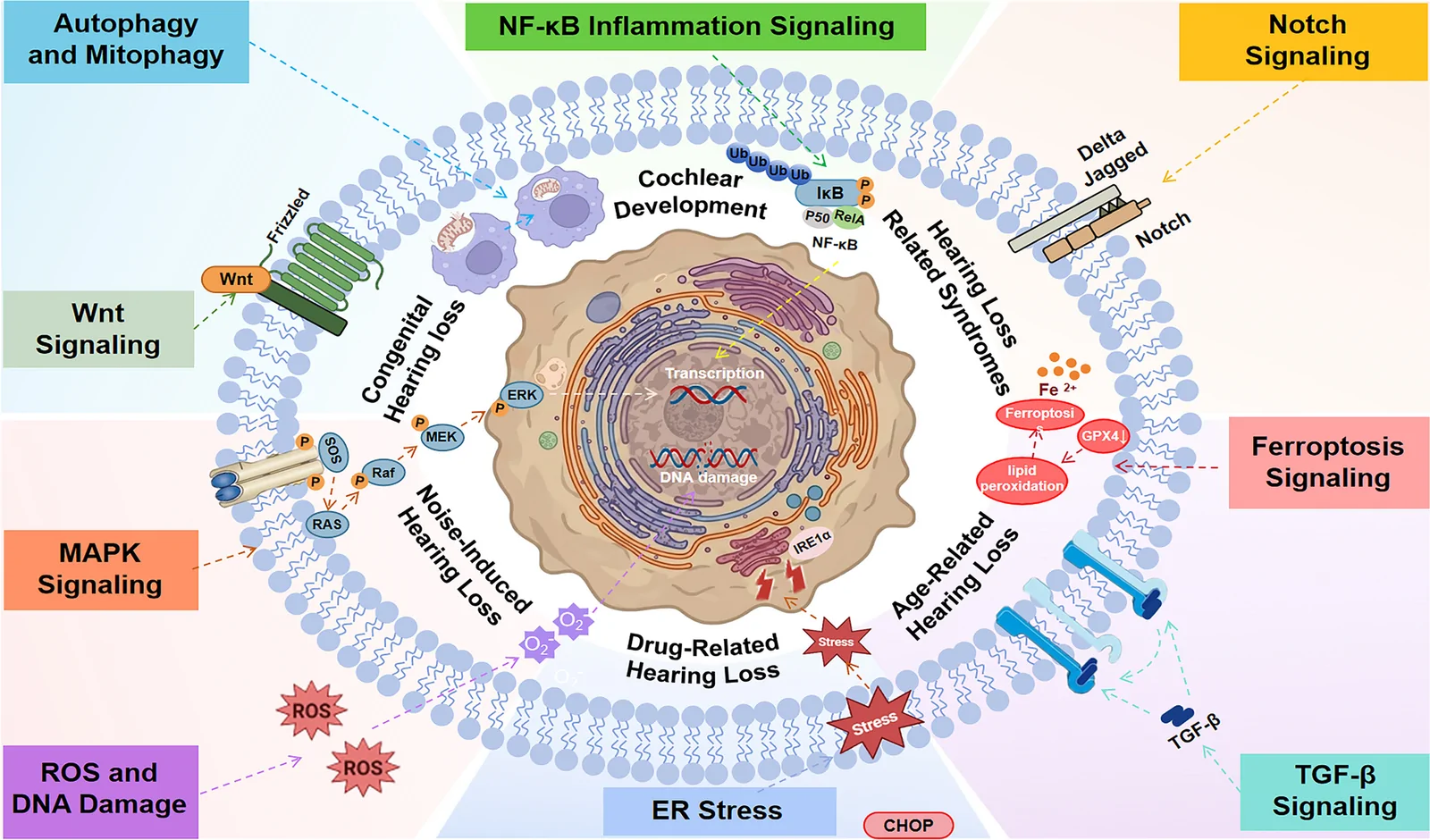
UPS critically regulates cochlear proteostasis in various aspects. During development, it correlates with Notch/Wnt/TGFβ/MAPK signaling to regulate cochlear tissue differentiation. Disruption of UPS can cause congenital deafness. In acquired SNHL (aging, noise and drugs), UPS integrates with multiple pathways including autophagy, mitophagy, oxidative stress, NF-κB signaling, ER stress, ferroptosis and DNA repair to maintain cochlear proteostasis

Many UPS-related molecules discussed above (Table 1) are involved in the pathogenesis of SNHL described. Most of them are involved in congenital hearing loss and ARHL, followed by NIHL and DRHL (Fig. 3). UPS serves as a central regulator of cochlear protein homeostasis by regulating several signaling and stress-responsive pathways (Fig. 4). During development, the UPS can finely regulate Notch, Wnt, TGFβ, and MAPK signaling pathways, thereby mediating the cell-cycle progression and arrest within specific developmental windows. These processes are essential for the differentiation of the prosensory region into hair cells and supporting cells, as well as regulating the establishment of planar cell polarity in hair cells. Dysregulation of UPS leads to developmental abnormalities and congenital deafness. In acquired hearing loss (aging, noise and drugs), the UPS closely integrates with multiple pathways including autophagy, mitophagy, oxidative stress, ERAD, and DNA damage repair, to coordinate protein degradation. Among them, mitochondrial quality control appears to be particularly important. The mitophagy mediated by the E3 ligase Parkin has been identified as a key protective mechanism across all three forms of SNHL [130, 165, 166]. Parkin dysfunction results in accumulation of damaged mitochondria, ATP depletion, and excessive production of ROS. Meanwhile, autophagic pathways, including both general autophagy and mitophagy, are also regulated by deubiquitinating enzymes such as CYLD [84] and UCHL1 [162]. Another major pathway is the NF-κB centered immune-inflammatory cascade, which is closely involved in most types of deafness. Multiple deubiquitinating enzymes (USP48 [111], USP53 [155, 156], UCHL1 [162]) and proteasome subunits PSMD12 [80, 81] participate in this pathway, highlighting the role of polyubiquitination in regulating inflammatory signaling in the cochlea. UPS also interacts with oxidative stress. GRAIL contributes to noise-induced oxidative stress and promotes hair cell apoptosis [151]. Keap1 maintains the expression of antioxidant genes at low level by degrading Nrf2 and causes elevated ROS in the aged cochlea [122]. The accumulated ROS can inhibit proteasome activity, which forms a vicious cycle. DNA damage repair is another classical pathway associated with apoptosis and aging. In the cochlea, it is primarily mediated by RAD6B and RNF8. Deficiency of these factors leads to accumulation of γ-H2AX, activation of the p53/p21 pathway, and cell-cycle arrest, resulting in accelerated cochlear degeneration [127, 129].

**Table 1:** Major roles of UPS related molecules in cochlear development or function, and in different types of hearing

**UPS related protein**	**Type**	**Pathogenic role** (gain/loss of function)	**Substrate**	**Related pathway**	**Function/Disease**
Huwe1 *(IIA)* [59]	HECT-type E3 ubiquitin ligase	Loss of function	Atoh1	• N/A	Cochlear development
Mdm2 *(IIA)* [62]	RING-type E3 ubiquitin ligase	Loss of function	p53	Notch signaling	Cochlear development
RBBP6 *(IIA)* [63, 64]	RING-type E3 ubiquitin ligase	Loss of function	p53	Notch signaling	Congenital hearing loss Adult-onset deafness
Mib1 *(IIA)* [69]	RING-type E3 ubiquitin ligase	Loss of function	intracellular domain of Delta	Notch signaling	Cell differentiation
Smurf1/2 *(IIA)* [71]	HECT-type E3 ubiquitin ligase	Loss of function	Prickle1	Non-canonical Wnt signaling	Planar polarity in hair cells
Trim71 *(IIA)* [75]	RING-type E3 ubiquitin ligase	Loss of function	N/A	TGFβ signaling	Congenital hearing loss
PSMC1 *(IIB)* [77]	Subunit of proteasome	Loss of function	/	N/A	Congenital hearing loss
PSMC3 *(I)* [78]	Subunit of proteasome	Loss of function	/	TCF11/Nrf1 signaling	Cochlear development
PSMD12 *(IIB)* [80]	Subunit of proteasome	Loss of function	/	NF-κB signaling*	Sensorineural or mixed hearing loss
Ubr1 *(IIB)* [86]	RING-type E3 ubiquitin ligase	Loss of function	N/A	Notch signaling	Johanson-Blizzard syndrome
Ubr3 *(I)* [87]	RING-type E3 ubiquitin ligase	Loss of function	Myosin II	Synaptic transmission	Usher syndrome
Ubr5 *(IIB)* [90]	HECT-type E3 ubiquitin ligase	Loss of function	SWI/SNF complex*	Notch signaling	Congenital hearing loss
Rma1 *(III)* [94]	RING-type E3 ubiquitin ligase	Loss of function	Pendrin	Endoplasmic reticulum stress	Pendred syndrome
RNF220* (IIB)* [95]	RING-type E3 ubiquitin ligase	Loss of function	Lamin B1 STAT1*	Nuclear lamina regulation	Sensorineural hearing loss
UBE3B *(IIB)* [98, 147]	HECT-type E3 ubiquitin ligase	Loss of function	BCKDK* Ppp3cc*	Metabolic Pathways*	Kaufman oculocerebrofacial syndrome Noise-related hearing loss
Stub1 (CHIP) *(I)* [102]	U-box E3 ubiquitin ligase	Loss of function	Diaph3	Notch signaling	Hearing impairment
BRWD3 *(I)* [105, 106]	Substrate-specificity factor of DDB1-CUL4 E3 ligase complex	Loss of function	KDM5	MAPK/Akt signaling	Congenital hearing loss Drug-related hearing loss
Keap1 *(IIA)* [122]	Cullin3-based E3 ubiquitin ligase adaptor	Gain of function	Nrf2	• Oxidative stress	Age-related hearing loss
UBA1 *(IIB)* [124]	E1 ubiquitin-activating enzyme	Loss of function	/	DNA damage*	Sensorineural hearing loss
RAD6B *(IIA)* [127]	E2 ubiquitin-conjugating enzyme	Loss of function	/	DNA damage	Age-related hearing loss
RNF8 *(IIA)* [128]	RING-type E3 ubiquitin ligase	Loss of function	H2A/H2AX	DNA damage	Age-related hearing loss Adult-onset deafness
Parkin *(IIA)* [134, 150, 163]	RING-type E3 ubiquitin ligase	Loss of function	MFN2	Mitophagy signaling	Congenital hearing loss Age-related hearing loss Noise-related hearing loss Drug-related hearing loss
Fbx2 *(I)* [140]	RING-type E3 ubiquitin ligase	Loss of function	Connexin26	E Endoplasmic reticulum stress*	Age-related hearing loss
Nedd4 *(IIA)* [142, 143]	HECT-type E3 ubiquitin ligase	Loss of function	Cx26	Ferroptosis signaling Endoplasmic reticulum stress	Age-related hearing loss
GRAIL *(IIA)* [151]	RING-type E3 ubiquitin ligase	Gain of function	SIRT1*	Immune inflammation Oxidative stress	Noise-related hearing loss
**Deubiquitination** **related protein**					
CYLD (I) [84]	Deubiquitinating enzyme	Loss of function	TRAF6*	Mitophagy signaling*	Hearing impairment
USP48 (formerly USP31) *(I)* [111]	Deubiquitinating enzyme	Loss of function	p65*	NF-κB signaling*	Congenital hearing loss
USP9X *(IIB)* [112]	Deubiquitinating enzyme	Loss of function	DVL2*	Canonical Wnt signaling Notch signaling	Axenfeld–Rieger syndrome
USP53 *(IIA)* [155, 156]	Deubiquitinating enzyme	Loss of function	Interact with tight junction scaffolding proteins TJP1 and TJP2	NF-κB signaling*	Noise-related hearing loss
UCHL1 *(IIA)* [162]	Deubiquitinating enzyme	Loss of function	N/A	NF-κB signaling Autophagy	Age-related hearing loss Drug-related hearing loss

*Means the substrates/related pathways were confirmed in other neuronal tissues or other neurodegenerative diseases rather than hearing loss

N/A means the substrates/related pathways were not mentioned or confirmed in both hearing loss and other neurodegenerative diseases

Here, we categorize the above-mentioned UPS-related genes/molecules into three tiers based on the strength of evidence.

I – Direct causality: Pathogenic variants of the genes/proteins or their substrates have been identified in human patients, with clear relation between the variant and the hearing loss phenotype. In addition, at least one animal model fully or partially simulates the human hearing phenotype or the developmental/degenerative phenotypes. These genes are considered most important to auditory function.

II – Strong associative causality:

IIA:Functional loss or knockout/knockdown studies in animal models are available, and the animal models exhibit clear hearing loss or inner ear developmental/degenerative phenotypes. However, human genetic evidence showing the relationship between the genes/molecules and auditory function is mainly limited.

IIB: Pathogenic variants of the genes/proteins or their substrates have been identified in human patients, with clear relation between the variant and the hearing loss phenotype. However, animal models validations showing the relationship between the genes/molecules and auditory function are mainly limited.

III – Inferred/correlative evidence: The current evidence is derived primarily from expression profiling, in vitro biochemical studies, or inferences from homologous genes/other tissues. Direct in vivo functional validation of causality between the protein and hearing loss is still incomplete.

Accumulation of misfolded proteins in the ER is another early event observed in multiple hearing loss models. In congenital hearing loss, mutant Pendrin protein is excessively degraded by Rma1-mediated ERAD. In contrast, *WFS1* mutation impairs ERAD function by destabilizing HRD1 in late-onset hearing loss. These results imply us that both excessive and insufficient ERAD activity can disrupt protein homeostasis and contribute to hearing loss.

These pathways do not operate in isolation. Instead, they form an interconnected and mutually amplifying network. For example, ROS stress can activate NF-κB [167] and MAPK [168] signaling pathways while inhibiting proteasome activity [169]. ER stress can trigger mitochondrial apoptosis [170] and ferroptosis [171] via calcium signaling. DNA damage activates p53, which may promote autophagy [172] or trigger apoptosis [173]. Overall, the UPS integrates developmental signalings, stress responses, and cell death decisions in different forms of SNHL.

## Tissue specificity of UPS-related molecules in cochlea

E3 ubiquitin ligases and DUBs exhibit pronounced tissue specificity across cochlear structures, particularly between hair cells and spiral ganglion neurons. This specificity is primarily manifested in two aspects: expression specificity and functional specificity.

### Expression specificity

Among all the UPS components discussed above, different molecules exhibit distinct expression patterns across inner ear structures. For example, UCHL1, a neuron-specific DUB, is highly expressed in spiral ganglion neurons but is nearly undetectable in hair cells [162, 174]. In contrast, Fbx2 is enriched in supporting cells and hair cells, whereas its expression in spiral ganglion neurons is relatively low [140]. USP53 is also highly expressed in cochlear hair cells (particularly outer hair cells) and parts of supporting cell [155]. Trim71 is predominantly localized in the sensory epithelium. It maintains the undifferentiated state of prosensory cells during embryonic development and enhances the regenerative potential of supporting cells postnatally [75]. These distinct expression patterns suggest that different cochlear cell types may depend on specific UPS components, which may partly explain their selective vulnerability in different forms of hearing loss.

### Functional specificity

Although many UPS-related molecules are broadly expressed in various structures in the inner ear, they show functional specificity during hearing formation or in different types of hearing loss. For example, CYLD is expressed in both hair cells and spiral ganglion neurons. However, it primarily regulates neurite outgrowth and the formation of synaptic connections during development, with limited effects on maintaining hair cell polarity or stereociliary structure [83]. USP48 is expressed in multiple structures of the developing cochlea, including spiral ganglion neurons, outer sulcus, interdental cells of the spiral limbus, stria vascularis, Reissner's membrane and in the transient Kolliker's organ. Nevertheless, *USP48* knockdown in zebrafish delays development of otic neurons with normal hair cell morphology, suggesting that its function is predominantly exerted in neurons [109]. These tissue- and cell type-specific functional divergence implies us that UPS-targeted therapeutic strategies about hearing loss should be designed with cell types to achieve more precise therapeutic modulation.

## UPS as a target for treatment in hearing loss

Currently, drug development directly targeting the ubiquitin–proteasome system for the treatment of SNHL is still at a very early exploratory stage. No drugs have yet entered the clinical trials. However, several in vivo animal studies have revealed that restoring proteostasis through the modulation of UPS can help to rescue various types of SNHL (Table 2). Additionally, growing therapeutic experience with UPS-targeted regulation has been accumulated in the field of central nervous system, which may provide important guidance for future translational studies in the auditory system. In this part, we systematically review the recent progress in modulating proteasome activity, gene therapy targeting specific E3 ubiquitin ligases and deubiquitinases through gene therapy, as well as restoring UPS function using chemical chaperones or small molecules in the auditory field.

**Table 2:** Molecules or drugs targeting UPS that show efficacy in treating hearing loss or alleviating degeneration of the cochlea

Molecules/Drugs	Intervention	In vivo/vitro	Related pathways	Efficacy
GRAIL [152]	Inhibition	In vivo	Reduce pro-inflammatory cytokines and chemokines	Improve noise/age -related hearing loss
PSMD11 [175]	Overexpression	In vivo	Enhance proteasome activity	Improve age-related hearing loss
Parkin [130, 165, 166]	Activation	In vivo	Enhance mitophagy	Improve noise/age/drug -related hearing loss
UCHL1 [174, 176]	Overexpression	In vitro	Inhibit NF-κB signaling and enhance proteasome activity	Inhibit HEI-OC1 and auditory cortex cell senescence
TRC051384 [177]	/	In vivo	Activate HSP70 to restore UPS function	Improve noise-related hearing loss
TUDCA [164, 178]	/	In vivo	Alleviate endoplasmic reticulum stress and restore protein ubiquitination levels	Improve drug-related hearing loss

### Regulation of proteasome activity: A double-edged sword

Studies have shown that proteasome activity gradually declines with aging. Thus, restoring or enhancing proteasome activity is recognized as a promising therapeutic approach for neurodegenerative diseases [179]. In AD, Aβ can bind to the 20S proteasome and impairs its activity. The activities of 20S and 26S proteasome are found reduced in the brains of aged mice, accompanied by decreased in 20S proteasome assembly [180]. Restoring proteasome function may represent a promising therapeutic strategy to mitigate neurodegeneration in AD [181]. Overexpressing the proteasome subunit PSMB5 in the neurons of mice to increase the proteasome content can rescue spatial learning and memory deficits [180]. Proteasome activator Blm10 and its human ortholog PA200 play a key role in regulating proteasome activity and assembly. Overexpression of Blm10/PA200 reduces α-synuclein aggregation via activation of the 20S proteasome in yeast and mammalian cells, which may have therapeutic potential in PD [182]. In the auditory system, upregulation of PSMD11 (a subunit of the 19S proteasome regulatory particle) can enhance proteasome activity and delay D-galactose induced aging of the auditory cortex [175].

By contrast, the inhibition of proteasome activity shows adverse effects on auditory organs. Lee et al. treat cultured organ of Corti with 5 μM proteasome inhibitor MG132 and observe hair cell damage mentioned above in part 3 [56]. However, this does not mean that higher proteasome activity is always beneficial. For instance, most X-linked non-syndromic hearing losses are caused by mutations in the *Pou3f4* gene, which is essential for hair cell survival [183]. Structurally abnormal Pou3f4 will be degraded by the proteasome system, resulting in transcriptional termination and hearing loss. Byung et al. show that treatment of 293 T cells with 25 μM MG132 significantly increases intracellular Pou3f4 expression in vitro [184]. Whether this approach can restore Pou3f4 expression to alleviate X-linked non-syndromic hearing loss requires further investigation. In mouse model of Pendred syndrome, the protein abundance of pathogenic pendrin variants is found significantly reduced, indicating accelerated protein degradation rather than defective protein synthesis. Incubation with 1–10 μM MG132 in vitro can rescue the expression of Pendrin, which represents a novel concept for the treatment of Pendred syndrome [185].

Proteasome inhibitors can also exert anti-tumor effects by blocking protein degradation and inducing apoptosis [186]. Thus, proteasome inhibition acts as a double-edged sword in therapeutic applications. Its potential benefits and risks must be carefully balanced, as the final outcome depends strongly on dose, duration, and cellular environment. Continuous exposure to high-concentration of proteasome inhibitors is often used to establish various models of central nervous system degeneration in animals. For example, intraperitoneal injection of MG-132 for 10 consecutive days after birth in C57BL/6 J mice leads to neuronal loss in the hippocampus, amygdala, and brainstem during adulthood, accompanied by reduced neurogenesis, memory impairment, motor dysfunction, and balance disorders [187]. Injection of the 20S proteasome inhibitor lactacystin into the substantia nigra of mice successfully induces pathological and behavioral features resembling PD, including dopaminergic neuron loss and α-synuclein aggregation, along with motor deficits [188]. However, many studies have found that transient or low concentration of proteasome inhibitors can induce neuroprotective effects and enhance neuronal resistance to the subsequent injury. In a classical mouse model of PD, subcutaneous injection of the proteasome inhibitor PSI can protect dopaminergic neurons from damage and reduce motor deficits through increasing protein ubiquitination levels [189]. Pretreatment with a low concentration of MG132 (1–5 μM) protects neuronal cell lines against endogenous ROS damage and exerts neuroprotective effects through the activation of NF-κB signaling pathway [190]. Mild MG132-mediated proteasome inhibition may also promote cell survival by inducing the expression of molecular chaperones, such as HSPs. These chaperones assist to process misfolded proteins, modulate ubiquitination balance and maintain proteostasis [191].

Therefore, current therapeutic strategies for various neurodegenerative diseases are no longer limited to simple "activation" or "inhibition", but have shifted to precise modulation of the UPS. The overall goal is to clear toxic proteins while avoiding the adverse effects of systemic proteasome inhibition.

### Therapeutic strategies targeting specific E3 ubiquitin ligases

In the auditory field, studies targeting the regulation of UPS-related enzymes to restore hearing are limited. Chuang et al. identify gene related to anergy in lymphocytes (GRAIL), a RING-type E3 ligase, as a molecule associated with hearing loss. GRAIL knockout mice show better auditory function after noise exposure or during the aging process through reducing pro-inflammatory cytokines and chemokines [151]. Another targeted E3 ligase is Parkin. The activation of Parkin-associated mitophagy pathway has been shown to ameliorate different types of SNHL in animal models [130, 165, 166]. In central nervous system, the Pink1/Parkin agonist ABBV-1088 is the only drug that has entered Phase I clinical trials for the treatment of Parkinson's disease [192]. However, a recent study publishes that this drug may not enhance mitophagy via the expected mechanism of the Pink1/Parkin pathway activation, but rather through mitochondrial toxicity. This finding raises significant questions about the safety and efficacy of Pink1/Parkin activators as a therapeutic strategy for such neurodegenerative disease [193]. Among the other E3 ubiquitin ligases discussed above in SNHL, most of them have not been verified in animal models to improve hearing function, but some of these ligases have been confirmed to delay neurodegeneration in the central nervous system diseases. For example, overexpression of Nedd4 in mouse primary cortical neuron can promote the clearance of mutant huntingtin protein (mHTT) in HD and reduce its neurotoxicity [194]. However, another member of the Nedd4 family—Nedd4L appears to exert an opposite effect in HD. Overexpression of Nedd4L leads to neuronal degeneration with reduced motor activities through disrupting mitochondrial function [195]. This discovery highlights the marked functional heterogeneity within the same E3 ligase family, suggesting that targeted treatments must be precise to distinguish subtypes. E3 ubiquitin ligase Stub1 (CHIP) mentioned above also acts as a co-chaperone. CHIP overexpression in APP/PS1 mouse brain significantly ameliorates the cognitive dysfunction of APP/PS1 mice in the Morris water maze, with decreased expression of both Aβ and phosphorylated tau [196].

In AD, promoting tau degradation is an effective strategy for the disease treatment. Sorafenib has been verified to attenuate cognitive deficits in PS19 tauopathy model mice through promoting the expression of the E3 ubiquitin ligase FBXW7, which binds tau and mediates tau degradation through UPS [197]. Based on target protein degradation by UPS, proteolysis-targeting chimeras (PROTACs) techniques have been invented and emerge as efficacious approaches in treating neurodegenerative diseases [198]. PROTACs consist of a ligand for the protein of interest (POI), a ligand for an E3 ubiquitin ligase and a linker. PROTACs facilitate the ubiquitylation and degradation of the POI through binding both the POI and the ligase [199]. In animal studies, targeted degradation of α-synuclein by arginine-based PROTACs for the treatment of PD [200] and selective degradation of misfolded SOD1 by biological PROTACs for the treatment of ALS [201] have demonstrated excellent therapeutic potential. As a result, these advances in the central nervous system provide valuable insights for future studies on UPS-targeted therapies for SNHL. Up till now, the PROTACs technology has undergone preliminary exploration in hearing loss. PROTAC-8 acts as a specific cyclin-dependent kinase 2 (CDK2) degrader with no effect on other closely related CDKs. In zebrafish models of cisplatin- and kainic acid-induced ototoxicity, PROTAC-8 is found to protect hair cells from their deleterious effects [202]. From this idea, specific PROTACs with upstream E3 ligases can be designed for the aggregation of pathogenic proteins such as over-activated Diaph3 in hearing loss.

### Therapeutic strategies targeting specific deubiquitinases

DUBs can reverse the degradation activity of the UPS by removing ubiquitin chains from substrate proteins. Targeting DUBs has emerged as a new therapeutic target for neurodegenerative diseases. In the auditory field, deubiquitinating enzyme UCHL1 mentioned above is specifically expressed in neurons and neuroendocrine cells [162]. UCHL1 overexpression can mitigate H2O2-induced oxidative injury and inhibit HEI-OC1 cell senescence through the NF-κB signaling pathway [174]. Moreover, in the D-galactose induced primary auditory cortex cells, increased UCHL1 expression can promote cellular ubiquitination levels and enhance proteasome activity [176]. The results show that UCHL1 may protect both peripheral auditory organs and central auditory cortex from ROS damage in the process of ARHL. In PD, UCHL1 can also regulate α-synuclein degradation and exert neuroprotective effects [203]. In addition, more DUBs especially USP family enzymes have been explored in PD, including USP7, USP8, USP9X, USP10, USP13, USP14, and USP30. Their inhibitors may have potential therapeutic effects in AD or PD [204, 205]. For example, the small molecule inhibitor Spautin‐1 can target USP10 and USP13 to alleviate cognitive deficits and memory impairment in mice after brain injury [206]. However, the upregulation of USP10 has potential value in the treatment of cancer through activating p53 signaling pathways [207]. Thus, targeting DUBs may have completely opposite effects in different diseases and thus requires careful selection.

### Therapeutic strategies to improve protein homeostasis and restore UPS function

Targeting molecular chaperones such as HSPs to improve protein folding, transportation, ubiquitination, and proteasomal degradation has become an active research area in neurodegenerative disease therapy. This strategy has been shown to protect the proteins from misfolding and abnormal aggregation [208]. In Alzheimer's disease, Tau is a high-affinity substrate of the HSP70/90 machinery, and E3 ubiquitin ligase CHIP effectively disassembles the machinery and targets Tau to proteasomal degradation [209]. In a mouse model of noise-induced hearing loss, treatment with the heat shock factor 1 (HSF1) activator TRC051384 restores UPS function by activating HSP70 expression, protecting spiral ganglion neurons and alleviating noise-induced hearing loss [177]. Wen et al. demonstrated that the chemical chaperone tauroursodeoxycholic acid (TUDCA) plays a key role in protein stabilization. It can effectively alleviate ER stress and restore protein ubiquitination levels, thereby reducing cisplatin- or gentamicin-induced hair cell apoptosis [164, 178]. In rats, both subcutaneous and intratympanic injection of TUDCA can significantly attenuate cisplatin-induced hearing loss [164, 210]. It can also reduce gentamicin-induced hearing loss in mice. Another small molecular chemical chaperone, 4-phenylbutyrate (4PBA), can also alleviate hearing loss and suppress hair cell death in *Cdh23*^erl/erl^ mutant mice. It exerts neuroprotective effects through relieving ER stress and maintaining proteostasis [211]. VRT534, a chemical chaperone previously used for the treatment of cystic fibrosis has recently been found to stabilize mutant Cx26 in vitro, suggesting its possibility for future treatment of hearing loss [212]. As a result, chemical chaperones are emerging as a potential therapeutic options for SNHL associated with protein misfolding.

## Conclusion

Strategies targeting the UPS face considerable challenges. The human genome encodes more than 600 E3 ubiquitin ligases. The same E3 ligases can recognize specific substrates across different tissues and exert diverse biological functions. In this review, only a limited number of molecules can be classified as Grade I, indicating that their association with hearing loss has been clearly established in human populations and further validated in animal models. Although many E3 ligases are known to function in the cochlea, their substrate profiles and regulatory networks remain poorly defined, hampering the identification of optimal therapeutic targets for SNHL. Moreover, modulation of proteasome activity has a pronounced double-edged effect. Excessive inhibition may promote neurodegeneration, whereas moderate inhibition can exert protective effects under certain conditions. Conversely, proteasome activation can enhance the clearance of toxic proteins but may also accelerate the degradation of proteins essential for normal cellular function. The same UPS component may even exert opposing effects in different tissues or diseases. For example, CYLD promotes neurite outgrowth within the cochlea but negatively regulates neuronal survival in PD. Different members of the Nedd4 family have contrasting roles in HD. This marked functional heterogeneity indicates that therapeutic strategies developed for the central nervous system cannot be directly translated into the auditory system.

Studies exploring the role of the UPS in the cochlea remain at an early stage, with substantial research gaps to be filled. Current mechanistic studies are largely descriptive. In this review, we propose a three-tier evidence classification for UPS related molecules to illustrate their relationship with hearing loss. Most of them are classified into Grade II or Grade III, indicating considerable gaps between clinical cases and laboratory findings. Many studies only focus on phenotypic observations, such as hearing loss following the deletion of a particular molecule, without defining its downstream substrates, catalytic kinetics, or regulatory networks in detail. Moreover, the specificity of UPS regulation among different cochlear cell types has not been systematically investigated. For example, UCHL1 is highly expressed in spiral ganglion neurons but is barely detectable in hair cells. Fbx2 is enriched in supporting cells, and USP53 is highly expressed in hair cells. However, the biological significance of these cell-specific expression patterns remains unclear. Single-cell ubiquitinomics of cochlear cell subtypes is needed to address these questions. In addition, reliable quantitative methods for assessing UPS activity and standardized evaluation frameworks need to be established.

In the future, single-cell multi-omics technologies may provide a critical approach to overcome current research barriers. A database can be established to systematically map UPS-related molecules and the dynamic changes of ubiquitination across different cochlear cell types, developmental stages, and models of SNHL. Such a database can help to clarify the expression patterns and tissue-specific substrate profiles of E3 ubiquitin ligases in hair cells, supporting cells, spiral ganglion neurons, and the stria vascularis. This will provide an essential foundation for precise therapies. In addition, molecules classified as Grade II or Grade III in this review should be further investigated using conditional knockout mouse models to validate their function in the cochlea.

Based on existing studies, clinical translation should focus on the development of distinct UPS-modulating strategies targeting to specific subtypes of SNHL. Congenital hearing loss often results from mutations in UPS-related genes that lead to loss of auditory function. Therefore, target protein supplementation strategy mediated by adeno-associated viral vectors or emerging inner-ear delivery systems may be a better approach. ARHL is associated with a progressive decline in UPS function, suggesting that proteasome activation may offer better therapeutic effects for preserving or restoring auditory function. Noise-induced and drug-induced hearing loss may involve compensatory UPS activation during acute stress followed by subsequent functional exhaustion, and thus may require stage-specific interventions. PROTACs have already undergone preliminary investigation in the auditory field. In the future, selective degradation of over-expressed or pathogenic proteins using specific E3 ligase can be designed for different forms of hearing loss. However, the successful application of PROTACs will depend on selecting suitable E3 ligases for the recognition of abnormal proteins. So it is necessary to explore the role of E3 ligases in different tissues of cochlea. The chemical chaperones TUDCA and 4-PBA preserve proteostasis by alleviating ER stress and have shown promising protective effects in the inner ear. Their combination with selective UPS modulators may provide a coordinated therapeutic strategy that couples upstream prevention of protein misfolding with the downstream clearance of aberrant proteins. The ultimate goal is to define the molecular pathology of UPS dysfunction across different subtypes of SNHL. With the aid of minimally invasive inner-ear delivery technologies, it may become possible to achieve individualized precision therapies guided by cell type–specific ubiquitinomics profiles in the cochlea.

## Data Availability

Not applicable.
